# Enhanced Store-Operated Ca^2+^ Signal of Small Intestinal Smooth Muscle Cells Accelerates Small Bowel Transit Speed in Type 1 Diabetic Mouse

**DOI:** 10.3389/fphys.2021.691867

**Published:** 2021-10-20

**Authors:** Fang Dai, Jizheng Guo, Yang Wang, Tian Jiang, Hongbo Chen, Ying Hu, Juan Du, Xianming Xia, Qiu Zhang, Bing Shen

**Affiliations:** ^1^Department of Endocrinology, The First Affiliated Hospital of Anhui Medical University, Hefei, China; ^2^School of Basic Medicine, Anhui Medical University, Hefei, China; ^3^Department of Obstetrics and Gynecology, Maternal and Child Health Hospital Affiliated to Anhui Medical University, Hefei, China; ^4^Digestive Medicine Center, Department of General Practice, The Seventh Affiliated Hospital, Sun Yat-sen University, Shenzhen, China

**Keywords:** type 1 diabetes, small bowel transit, store-operated Ca^2+^ entry, Orai1, Ca^2+^-activated K^+^ channel, small intestinal smooth muscle

## Abstract

**Aims:** The underlying mechanism of diabetic enteropathy, a common complication of type 1 diabetes, remains unclear. Store-operated Ca^2+^ entry (SOCE) is a ubiquitous type of Ca^2+^ influx involved in various cellular functions. Here, we show that SOCE-related stromal interaction molecule 1 (STIM1) and Orai1 participate in inappropriate cellular Ca^2+^ homeostasis, augmenting agonist-induced small intestinal smooth muscle contraction and small bowel transit speed in a mouse model of type 1 diabetes.

**Methods and Results:** We used small interfering (si)RNA to suppress STIM1 and Orai1 proteins, and employed intracellular Ca^2+^, small intestinal contraction and intestinal transit speed measurement to investigate the functional change. We found that SOCE activity and Orai1 and STIM1 expression levels of small intestinal smooth muscle were significantly increased in cells cultured in high glucose medium or in diabetic mice. Gastrointestinal transit speed and SOCE-mediated contractions were markedly increased in diabetic mice; Knocking down Orai1 or STIM1 with siRNA rescued both alterations in diabetic mice. However, the Orai1-large conductance Ca^2+^-activated K^+^ (BK_Ca_) channel interaction was decreased in diabetic mice, and suppressing Orai1 expression or inhibiting the BK_Ca_ channel increased agonist-induced small intestinal contractions in normal mice.

**Conclusion:** We concluded that the increased SOCE caused by excessive STIM1 and Orai1 expression and decreased Orai1-BK_Ca_ interaction augmented small intestinal smooth muscle contraction and accelerated small bowel transit speed in diabetic mice. This finding demonstrates a pathological role for SOCE in diabetic enteropathy and provides a potential therapeutic target for diabetic enteropathy.

## Introduction

Type 1 diabetes is an immune-mediated metabolic disease characterized by insulin deficiency and glucose fluctuation accompanied by diverse complications (Cheng and Yilmaz, 2015). Diabetic gastrointestinal disorders are common complications, including diabetic gastroparesis, and intestinal enteropathy (Yarandi and Srinivasan, 2014). Many studies have reported that diabetic gastroparesis is caused by abnormal electrophysiological activity, impaired gastric enteric neurons, and incongruous muscle activity (Stevens et al., 2013; Koch and Calles-Escandon, 2015). However, studies examining diabetic enteropathy are relatively rare. Currently, diabetic enteric complications in type 1 diabetes, mainly constipation and diarrhea, are commonly considered a manifestation of symptomatic diabetic autonomic neuropathy (Yarandi and Srinivasan, 2014). Accumulated evidence shows that neurodegeneration of the myenteric plexus (Anitha et al., 2006) and loss of mucosal nerve fibers (Selim et al., 2010) are basic factors. Electric stimulation caused by luminal contents relay neuronal activity to generate muscle responses; however, nervous dysfunction results in a mild increase of cholinergic contractions, decrease of noradrenaline, and attenuated inhibitory neurotransmission in the ileum of mice with type 1 diabetes (Domenech et al., 2011; Uranga-Ocio et al., 2015). In addition, degeneration of the interstitial cells of Cajal and reduced association with enteric neurons lead to decreased electrical activity (Iwasaki et al., 2006). Although Ca^2+^ is an essential participant in smooth muscle contraction, the changes in cellular Ca^2+^ homeostasis of the small intestinal smooth muscle (SISM) in type 1 diabetes is still not entirely clear.

Ca^2+^ plays a critical role in numerous physiological functions. Store-operated Ca^2+^ entry (SOCE) is a ubiquitous mechanism of Ca^2+^ influx in many cell types (Putney, 2011). With the development of RNA interfering techniques, two major SOCE-related components were discovered: stromal interaction molecule 1 (STIM1) and Orai1 (Feske et al., 2006; Soboloff et al., 2006). The N-terminus of STIM1 acts as a Ca^2+^ sensor located in the lumen of the endoplasmic reticulum (ER)/sarcoplasmic reticulum (SR). Upon Ca^2+^ depletion of the ER/SR, STIM1 aggregates and translocates to ER-plasma membrane junction regions and then activates Orai1, which is an ion channel formed by four transmembrane spanning protein subunits (Liou et al., 2005; Prakriya et al., 2006; Soboloff et al., 2006). When agonists act on G protein-coupled receptors (GPCR), GPCR signaling through the Gaq activates phospholipase C (PLC) to drive both inositol triphosphate (IP_3_) and diacylglycerol (DAG) production, IP3 acts on IP_3_ receptors, causing Ca^2+^ release from the ER/SR and depletion of internal Ca^2+^ stores. Growing evidence indicates an association of altered SOCE with various diabetic complications. Enhanced SOCE in glomerular mesangial cells (Chaudhari et al., 2014; Chaudhari and Ma, 2016), vascular endothelium (Tamareille et al., 2006; Daskoulidou et al., 2015), and platelets (Chaudhari and Ma, 2016) contributes to diabetic nephropathy and vasculopathy. Attenuated SOCE, found in retinal microvascular smooth muscle (Curtis et al., 2003) of diabetic rats, reduces vessel contractile responses or may be a compensatory response to avoid over-reactive contraction in diabetes. However, the pathological role of SOCE in SISM in type 1 diabetes remains unknown.

Therefore, in the present study, we used a mouse SISM cell line (MUS-M1) to investigate changes of SOCE in a high glucose environment *in vitro*. We also examined the pathological role of the SOCE-mediated Ca^2+^ signal in small intestine transit speed *in vivo* in a mouse model of type 1 diabetes.

## Materials and Methods

### Materials

Thapsigargin (TG), carbachol (CCh), and adenosine triphosphate (ATP) were obtained from Calbiochem. Iberiotoxin (IbTX) and streptozotocin (STZ) were purchased from Sigma-Aldrich. Anti-STIM1 (sc-68897) and anti-Orai1 (sc-68895) primary antibodies were purchased from Santa Cruz Biotechnology. The anti-BK_Ca_ channel (APC-107) primary antibody was purchased from Alomone Lab. Specific small interfering (si)RNA for mouse STIM1 (5′-UACAGUGGCUCAUUACGUA-3′) and Orai1 (5′- GCCAUAAGACGGACCGGCA-3′) (Dietrich et al., 2007; Potier et al., 2009) and scrambled siRNA (5′-ACGCGUAACGCGGGAA UUU-3′) were designed and synthesized by Biomics Company.

### Cell Culture and siRNA Transfection

MUS-M1, a mouse SISM cell line, was purchased from the Kunming Cell Bank of the Chinese Academy of Sciences and cultured in Dulbecco’s Modified Eagle’s Medium supplemented with 100 U/mL penicillin, 100 μg/mL streptomycin, and 10% fetal bovine serum. Only sub-passage 3–10 MUS-M1 cells were used in the present study. For the group with normal glucose (NG) levels, the culture medium contained 5.6 mM glucose and 20 mM α-mannitol as an osmotic control. In the group exposed to high glucose (HG), the medium contained 25 mM glucose (Chaudhari et al., 2014). The medium was replaced every other day. Specific siRNAs against mouse STIM1 and Orai1 or scrambled control siRNA were transiently transfected into MUS-M1 cells using Lipofectamine 2000 reagent (Invitrogen) according to the manufacturer’s protocol. Cells were harvested for the following experiments 36 h after siRNA transfection.

### Intracellular Ca^2+^ Measurement

The cytosolic Ca^2+^ concentration ([Ca^2+^]_i_) was measured as described previously (Chen et al., 2016). Briefly, MUS-M1 cells were plated at 1 × 10^5^ per well on glass coverslips in 12-well culture plates as described above. Cells were loaded with a fluorescent Ca^2+^ ion indicator Fluo-8/AM (6 μmol/L) and 0.02% pluronic F-127 at 37°C for 30 min. The Ca^2+^ stores in MUS-M1 cells were depleted using 2 μmol/L TG or 100 μmol/L CCh for 10 min in Ca^2+^-free saline solution, which contained (in mmol/L) 140 NaCl, 5 KCl, 1 MgCl_2_, 10 glucose, 0.2 EGTA, and 5 HEPES at pH 7.4. The Ca^2+^ influx was initiated by applying 2 mmol/L extracellular Ca^2+^. The fluorescence signal was recorded using a Nikon fluorescence microscope with 488-nm excitation and 515-nm long pass emission wavelengths. Changes in the peak value of cytosolic [Ca^2+^]_i_ were displayed as the ratio of fluorescence intensities relative to the baseline intensity before the application of extracellular Ca^2+^ (F_1_/F_0_). Each data point is an average of 20–30 cells.

### Western Blotting and Co-immunoprecipitation

Western blotting and co-immunoprecipitation assays were performed as described in our previous study (Chen et al., 2016). Proteins were extracted from the lysates of MUS-M1 cells and the SISM tissue from diabetic and normal mice with detergent extraction buffer, which contained 1% Nonidet P-40, 150 mmol/L NaCl, and 20 mmol/L Tris–HCl at pH 8.0, plus protease inhibitor cocktail tablets. For SISM tissue, the peripheral adipose tissue and intestinal villi was removed. The extracts were fractionated by 12% SDS-PAGE and then transferred to polyvinylidene difluoride membranes. The membrane was blocked with 5% non-fat milk diluted by PBST for 1 h at room temperature and incubated at 4°C overnight with anti-Orai1 and anti-STIM1 primary antibodies. The next day, the membrane was washed three times and then incubated with a horseradish peroxidase-conjugated secondary antibody. The signal for each protein was detected using an ECL system. The optical densities of the protein bands were normalized to that of α-actin analyzed within the same lane and presented as the percentage of the optical density.

### Induction of Type 1 Diabetes

All animal experiments were performed according to the guidelines presented in NIH publication no. 8523 and approved by the Experimentation Ethics Committee of Anhui Medical University. Five-week-old Kunming mice (male) were provided by the Experimental Animal Center of Anhui Medical University and randomly divided into two groups: STZ and age-matched controls. Both groups were fed regular chow and given free access to tap water. STZ (6 mg/mL) was dissolved in 50 mM sodium citrate buffer (pH 4.4) before the injection. As previously described, mice in the STZ group were injected with STZ (40 mg/kg) intraperitoneally for five consecutive days to induce type 1 diabetes (Furman, 2015). Mice in the control group received injections of acetate buffer. Plasma glucose levels were determined with a One Touch Blood Glucose Monitoring System (LifeScan, Inc., United States). Mice with fasting plasma glucose levels higher than 11.1 mmol/L were selected for use.

### *In vivo* siRNA Injections

Diabetic mice were randomly separated into three groups. Each mouse received two injections every 3 days of Orai1 siRNA, STIM1 siRNA, or scrambled control siRNA. Based on previous studies (Filleur et al., 2003; Sang et al., 2018), all siRNA (in 50 μL of saline in each case) injections were administered intraperitoneally (125 μg/kg/day). The mice were used 3 days after the second siRNA treatment. In our intestinal smooth muscle cell culture studies using MUS-M1 cells, siRNA-mediated suppression of either Orai1 or STIM1 by 36% was sufficient to inhibit SOCE significantly. Based on these findings, we set a threshold of a minimum of 36% reduction in Orai1 or STIM1 protein within the intestinal wall as an inclusion criterion when assessing mice treated with siRNA *in vivo* via i.p. injections. With our approach, 80% of the animals injected with siRNA met this criterion and were analyzed as described.

### Intestinal Transit Speed

The small intestinal transit speed experiment was performed as described elsewhere (Yuece et al., 2007). Briefly, after an overnight fast (water *ad libitum*), a charcoal meal marker (10% charcoal suspension in 5% gum arabic, 0.1 mL per 10 g body weight) was freshly prepared by dispersion. The mixture was administered orally to assess upper gastrointestinal transit speed. After 20 min, the mice were euthanized using an overdose of CO_2_ gas, and the intestines were immediately isolated. The distance traveled by the marker was measured (in centimeters). The results of the intestinal transit speed experiment are shown as a percentage of the total length of the mouse small intestine, from the pylorus to the terminal ileum.

### Preparation of Small Intestine Segment and Tension Measurement

Small intestine segment preparation and tension measurements were performed as described elsewhere (Montgomery et al., 2016). Briefly, the mice were killed by an overdose of CO_2_ gas. The small intestine was quickly dissected and placed in Krebs-Henseleit solution containing (in mmol/L) 118 NaCl, 4.7 KCl, 2.5 CaCl_2_, 1.2 KH_2_PO_4_, 1.2 MgSO_4_, 25.2 NaHCO_3_, and 11.1 glucose, pH 7.4 at room temperature. The peripheral adipose tissue was removed. The small intestine was cut into segments 2 cm in length. One end of the tissue was fastened to a hook on the bottom of a glass organ bath. The other end was connected to an isometric force transducer, which was connected to an amplifier. The bubbling O_2_ should mix the solution. The tension signal was recorded and analyzed by a data acquisition and analysis system (BL-420E+, Chengdu Technology & Market Corp.). The segments were placed in 5 mL organ baths containing Krebs solution at 37°C and continuously bubbled with a gas mixture of 95% O_2_ and 5% CO_2_ to maintain pH at 7.4. After equilibrating for 60 min with 1 g passive tension, the small intestine segments were activated by two applications of high-K^+^ solution, containing 60 mmol/L K^+^, prepared by replacing NaCl with an equimolar amount of KCl. Next, the contractile response to CCh (100 μmol/L) or Ca^2+^ (2 mmol/L) was determined. Detailed group treatments are described in the section “Results.” For the IbTX group, IbTX (50 nmol/L) was added to Krebs-Henseleit solution for 20 min pretreatment.

### Statistical Analysis

Data are presented as means ± SEM. Student’s unpaired *t*-test was used to analyze the difference between two groups with SigmaPlot 12.5 software. A value of *P* < 0.05 was considered statistically significant.

## Results

### Effect of a High Glucose Environment on Store-Operated Ca^2+^ Entry in MUS-M1 Cells

**FIGURE 1:**
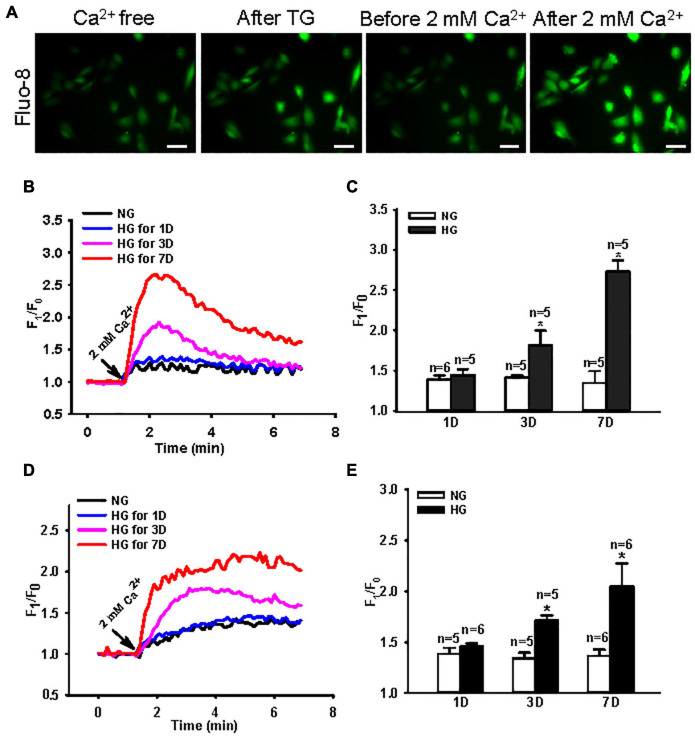
Effect of high glucose (HG) culture medium on store-operated Ca^2+^ entry (SOCE) in MUS-M1 cells. **(A)** SOCE was evoked by a classic “Ca^2+^ added back” protocol. Images showing fluorescence intensity indicating the change of transient intracellular Ca^2+^ concentration ([Ca^2+^]_i_). Scale bars, 10 μm. Representative traces **(B,D)** and summarized data **(C,E)** showing the change in [Ca^2+^]_i_ (SOCE) evoked by extracellular application of 2 mM Ca^2+^ after the treatment of 2 μM thapsigargin (TG) **(B,C)** or 100 μM carbachol **(D,E)** for 20 min in Ca^2+^-free solution to deplete internal Ca^2+^ stores in MUS-M1 cells cultured in normal glucose (NG, 5.6 mM D-glucose + 20 mM α-mannitol) or HG (25 mM D-glucose) medium for 1, 3, and 7 days. Values are shown as the mean ± SEM (*n* = 5–6); *P < 0.05, NG vs. HG on the same day.

Store-operated Ca^2+^ entry is a crucial Ca^2+^ signal that initiates many cellular biological processes (Putney, 2011; Chaudhari and Ma, 2016). Several cell types cultured in HG medium mimicking diabetic hyperglycemia *in vitro* show enhanced SOCE (Tamareille et al., 2006; Chaudhari et al., 2014; Daskoulidou et al., 2015). To determine the effect of an HG environment on SOCE in intestinal smooth muscle cells, we used Ca^2+^ imaging and measured the change in [Ca^2+^]_i_ evoked by SOCE in NG- and HG-cultured MUS-M1 cells. The Ca^2+^ store in MUS-M1 cells was depleted by 2 μM TG (a Ca^2+^ pump inhibitor in the ER) and 100 μM CCh (a muscarinic receptor activator). SOCE was initiated by the re-addition of 2 mM Ca^2+^ using a classic “Ca^2+^ added back” protocol. MUS-M1 cells were cultured in NG and HG media for 1, 3, and 7 days. The SOCE evoked by TG and CCh was significantly increased in HG-cultured cells at 3 and 7 days, but not at 1 day, compared with that in NG-cultured cells at 1, 3, and 7 days (Figure 1). By contrast, TG- and CCh-induced Ca^2+^ transients in Ca^2+^-free solution were not significantly changed between NG- and HG-cultured cells (Supplementary Figure 1). These results indicate that a HG environment enhances SOCE in MUS-M1 cells.

### Role of Orai1 and Stromal Interaction Molecule 1 in Enhanced Store-Operated Ca^2+^ Entry in High Glucose-Cultured MUS-M1 Cells

**FIGURE 2:**
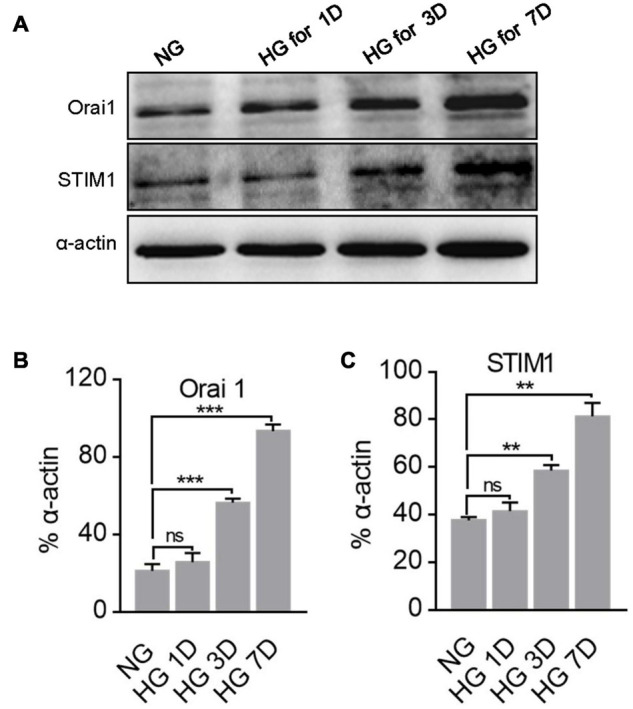
Effect of high glucose (HG) culture medium on Orai1 and STIM1 protein expression in MUS-M1 cells. Representative immunoblotting images **(A)** and summarized data **(B,C)** showing the expression levels of Orai1 **(B)** and STIM1 **(C)** proteins in MUS-M1 cells cultured in normal glucose (NG, 5.6 mM D-glucose + 20 mM α-mannitol) or HG (25 mM D-glucose) medium for 1, 3, and 7 days. Alpha-actin was used as a loading control. Values are shown as the mean ± SEM (*n* = 3); **, and *** denote *P* < 0.01, and *P* < 0.001, respectively. NS means non-significant, NG vs. HG on the same day.

Orai1 and STIM1 are two essential components in SOCE. Therefore, changes in Orai1 and STIM1 protein expression may affect the intensity of SOCE. Thus, we examined the expression profiles of Orai1 and STIM1 proteins in NG- and HG-cultured MUS-M1 cells. We found that Orai1 and STIM1 expression levels were significantly enhanced in HG-cultured cells on 3 and 7 days, but not 1 day, compared with those in NG-cultured cells on 1, 3, and 7 days (Figure 2).

**FIGURE 3:**
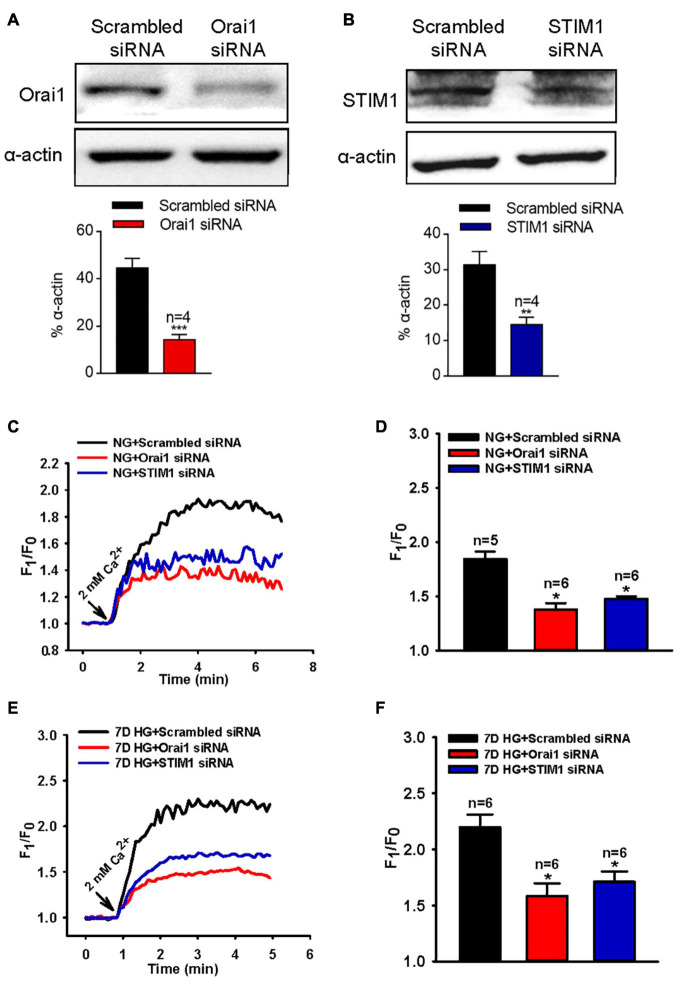
Effect of Orai1 and STIM1 siRNAs on store-operated Ca^2+^ entry (SOCE) in MUS-M1 cells. **(A,B)** Representative immunoblotting images showing Orai1 and STIM1 expression levels in MUS-M1 cells transfected with Orai1 siRNA **(A)**, STIM1 siRNA **(B)**, or scrambled control siRNA. **, and *** denote *P* < 0.01, and *P* < 0.001, respectively. Representative traces **(C,E)** and summarized data **(D,F)** showing the alterations in [Ca^2+^]_i_ (SOCE) evoked by extracellular application of 2 mM Ca^2+^ after the treatment of 100 μM carbachol for 10 min in Ca^2+^-free solution to deplete internal Ca^2+^ stores in MUS-M1 cells cultured in normal glucose (NG, **C,D**, 5.6 mM D-glucose + 20 mM α-mannitol) or high glucose (HG, **E,F**, 25 mM D-glucose) medium for 7 days. Alpha-actin was used as a loading control. Values are shown as the mean ± SEM (*n* = 5–6); *P < 0.05, scrambled siRNA vs. Orai1 or STIM1 siRNA transfections.

To further identify the role of Orai1 and STIM1 in the SOCE of MUS-M1 cells, we used an RNA interfering technique to suppress Orai1 and STIM1 protein expression. We found that compared with the scrambled siRNA control, Orai1- and STIM1-specific siRNAs knocked down Orai1 and STIM1 expression, respectively, in MUS-M1 cells (Figures 3A,B). In addition, our [Ca^2+^]_i_ measurement data indicated that compared with the scrambled siRNA control, Orai1- and STIM1-specific siRNAs significantly reduced CCh-evoked SOCE in NG- or HG-cultured (for 7 days) MUS-M1 cells (Figures 3C–F). However, CCh-induced Ca^2+^ transients in Ca^2+^-free solution were not significantly changed among scrambled, STIM1, and Orai1 siRNA-transfected cells (Supplementary Figures 2A,B). Pre-incubation with BTP-2 (20 μM) for 20 min significantly inhibited the SOCE of MUS-M1 cells on the 7 days in NG and HG-cultured (Supplementary Figures 2C,D). This result is consistent with the result using specific siRNA. Taken together, these data indicate that Orai1 and STIM1 are two important proteins participating in SOCE, and the increased Orai1 and STIM1 expression may amplify the SOCE intensity in HG-cultured MUS-M1 cells.

### Role of Orai1 and Stromal Interaction Molecule 1 in Small Intestine Contraction and Gastrointestinal Transit Speed in Diabetic Mice

**FIGURE 4:**
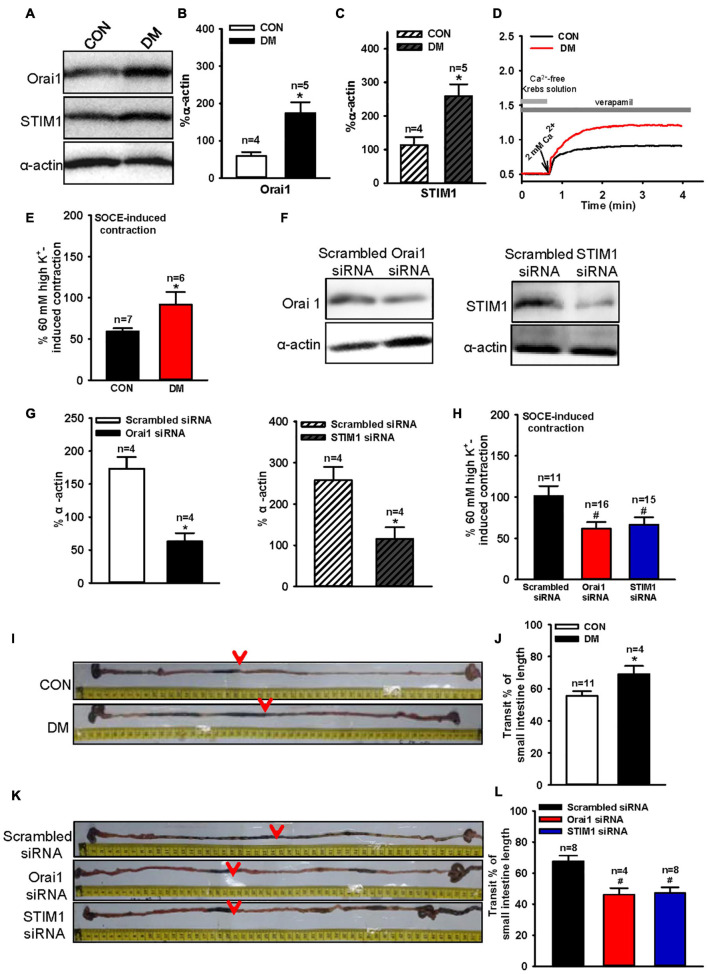
Orai1 and STIM1 protein expression levels, store-operated Ca^2+^ entry (SOCE)-mediated mouse small intestinal longitudinal contraction and intestinal transit time alterations in diabetic mice and the effect of Orai1 and STIM1 siRNA transfections. Representative immunoblotting images **(A)** and summarized data **(B,C)** showing the expression levels of Orai1 **(A,B)** and STIM1 **(A,C)** proteins in small intestinal smooth muscle cells of age-matched control (CON) and diabetic (DM) mice. Alpha-actin was used as a loading control. Values are shown as the mean ± SEM (*n* = 4–5); *P < 0.05, age-matched control vs. diabetic mice. Mouse small intestine segments were precontracted by the application of 100 μM carbachol in Ca^2+^-free Krebs solution to evoke SOCE. Then, re-addition of 2 mM Ca^2+^ induced longitudinal contractions of small intestine segments. Representative traces **(D)** and summarized data **(E,F)** showing SOCE-mediated small intestinal longitudinal contraction in age-matched control (CON) and diabetic (DM) mice **(D,E)**. Representative immunoblotting images and summarized data showing Orai1 and STIM1 expression levels in diabetic mice small intestinal smooth muscle cells transfected with Orai1 siRNA or STIM1 siRNA or scrambled control siRNA. Alpha-actin was used as a loading control **(F,G)**. Values are shown as the mean ± SEM (*n* = 4); *P < 0.05, scrambled siRNA vs. Orai1 or STIM1 siRNA transfection. SOCE-mediated small intestinal longitudinal contraction in diabetic mice transfected with scrambled, Orai1 or STIM1 siRNA **(H)**. Values are shown as the mean ± SEM (*n* = 6–16); *P < 0.05, age-matched control vs. diabetic mice; ^#^*P* < 0.05, scrambled siRNA vs. Orai1 or STIM1 siRNA. Representative images **(I,K)** and summarized data **(J,L)** showing intestinal transit distance in age-matched control (CON) and diabetic (DM) mice **(I,K)**, or diabetic mice transfected with scrambled, Orai1, or STIM1 siRNA **(J,L)**. The dark charcoal marker appeared as indicated (red arrows). Values are shown as the mean ± SEM (*n* = 4–11); *P < 0.05, age-matched control vs. diabetic mice; ^#^*P* < 0.05, scrambled siRNA vs. Orai1 or STIM1 siRNA.

Type 1 diabetes commonly induces gastrointestinal dysfunction, including constipation and diarrhea. The results above indicated that culturing cells in an HG environment increased Orai1 and STIM1 expression levels. Therefore, we generated type 1 diabetic mice by injecting them with STZ intraperitoneally, and investigated Orai1 and STIM1 protein expression in SISM. We found that Orai1 and STIM1 expression levels in the SISM of diabetic mice were significantly increased compared with those in age-matched controls (Figures 4A–C). Ca^2+^ is crucial for inducing smooth muscle contractions, and Orai1 and STIM1 mediate Ca^2+^ influx. Thus, altered Orai1 and STIM1 expression may affect the SOCE-mediated contraction of the small intestine. We next used 100 μM CCh to evoke SOCE in fresh-isolated small intestine segments bathed in Ca^2+^-free Krebs solution, in which 0.2 mM EGTA was used to chelate residual Ca^2+^ ions in the bath, and 1 μM verapamil to inhibit voltage-dependent Ca^2+^ channels (VDCCs). Re-adding 2 mM Ca^2+^ induced longitudinal contractions of the small intestine segment. Because VDCCs were inhibited by verapamil, any contraction induced by re-adding Ca^2+^ would be due mainly to the SOCE-mediated Ca^2+^ influx. These results indicated that SOCE-mediated small intestine segment contractions were significantly increased in diabetic mice compared with those in control mice (Figures 4D,E). However, 60 mM (high) K^+^-induced and CCh-induced contractions were not altered in diabetic mice (Supplementary Figures 3A,C). Additionally, hematoxylin and eosin (HE) staining showed no obvious difference in the thickness of the smooth muscle layer of the small intestine between the mouse groups (Supplementary Figure 4).

To verify the role of Orai1 and STIM1 in the enhanced small intestine contractions mediated by SOCE, we intraperitoneally injected Orai1- or STIM1-specific siRNA into diabetic mice to suppress Orai1 or STIM1 expression, respectively, in SISM cells. After siRNA treatment for 6 days (two injections every 3 days), western blotting analysis showed that Orai1 and STIM1 expression levels were significantly reduced in the SISM of diabetic mice (Figures 4F,G). Our contraction experiments indicated that compared with scrambled siRNA transfection, Orai1- or STIM1-specific siRNA transfection largely suppressed the SOCE-mediated small intestine contraction in diabetic mice (Figure 4H) but did not affect 60 mM (high) K^+^-induced contraction caused by voltage-dependent Ca^2+^ channel-mediated Ca^2+^ influx in Krebs solution or CCh-induced contraction in Ca^2+^-free solution, in which CCh-induced contraction is induced by Ca^2+^ release from Ca^2+^ store (Supplementary Figures 3B,D).

Small intestinal longitudinal contractions are correlated with gastrointestinal transit speed (Grider, 2003). Therefore, we investigated gastrointestinal transit speed in another group of diabetic mice. Gastrointestinal transit speed was significantly increased in diabetic mice compared with that in age-matched controls (Figures 4I,J). Additionally, transfections with Orai1- and STIM1-specific siRNAs significantly decreased the gastrointestinal transit speed of diabetic mice (Figures 4K,L). These results suggest that increased Orai1 and STIM1 expression may contribute to the enhancement of agonist-induced small intestine contraction and gastrointestinal transit speed.

### Role of the Orai1-Large Conductance Ca^2+^-Activated K^+^ Channel Interaction in Agonist-Induced Small Intestine Contraction

**FIGURE 5:**
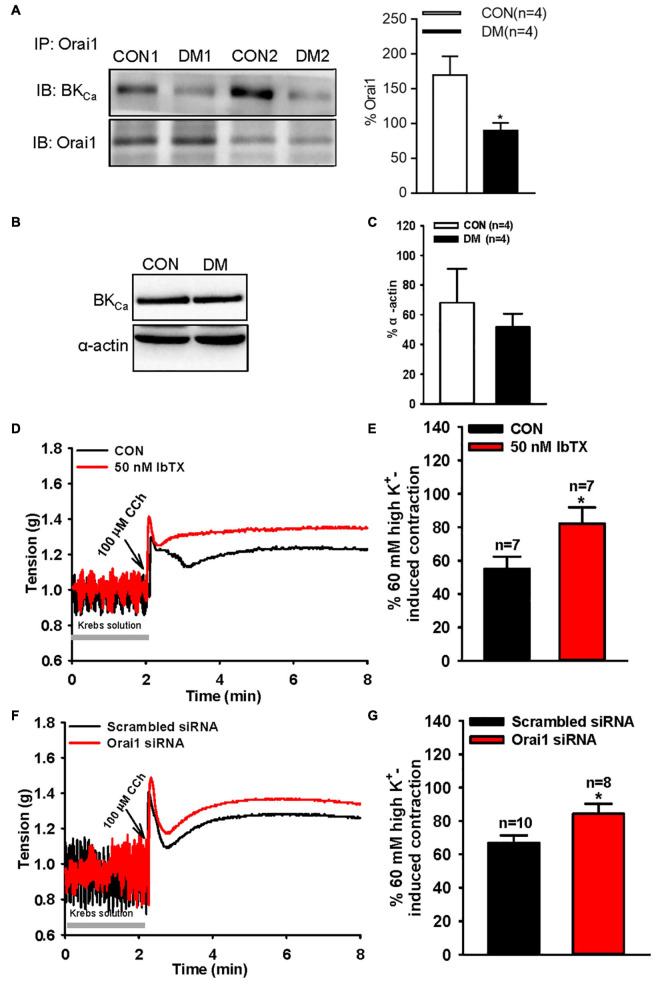
Orai1-BK_Ca_ interaction and BK_Ca_ channel protein expression level alterations in diabetic mouse small intestinal smooth muscle cells and role of store-operated Ca^2+^ entry (SOCE) and the BK_Ca_ channel in carbachol (CCh)-induced mouse small intestinal longitudinal contraction. **(A)** Representative paired images and summarized data showing co-immunoprecipitation followed by immunoblotting (IB) with anti-BK_Ca_ channel or anti-Orai1. Proteins from small intestinal smooth muscle of age-matched control (CON) or diabetic (DM) mice were immunoprecipitated with anti-Orai1 antibody; *n* = 4 experiments. Representative images **(B)** and summarized data **(C)** showing the expression level of the BK_Ca_ channel protein. Alpha-actin was used as a loading control. Values are shown as the mean ± SEM (*n* = 4); *P* > 0.05, age-matched control vs. diabetic mice. Representative traces **(D,E)** and summarized data **(E,G)** showing 100 μM CCh-induced mouse small intestine contractions. The segments of the small intestine were pretreated with 50 nM IbTX for 20 min **(D,E)** or transfected with scrambled or Orai1 siRNA **(F,G)** by intraperitoneal injections. Values are shown as the mean ± SEM (*n* = 7–10); *P < 0.05, control (CON) vs. IbTX treatment, or scrambled siRNA vs. Orai1 siRNA.

Our recent studies have demonstrated that Orai1 associates with Ca^2+^-activated K^+^ channels (for example, the small conductance Ca^2+^-activated K^+^ channel 3 and BK_Ca_ channel) to form signal complexes regulating smooth muscle contraction (Song et al., 2015; Chen et al., 2016). Here, we also investigated the interaction of Orai1 and the BK_Ca_ channel in SISM. We first used co-immunoprecipitation assays followed by immunoblotting and found that Orai1 pulled down the BK_Ca_ channel from mouse SISM (Figure 5A). We then used tension measurements to examine the function of the Orai1-BK_Ca_ complex in SISM. In normal mice, our data showed that IbTX (a specific inhibitor of BK_Ca_ channels, 50 nM) and Orai1 siRNA transfection (Supplementary Figure 5) significantly increased 100 μM CCh-induced small intestinal longitudinal contractions (Figures 5D–G), but did not affect 60 mM (high) K^+^-induced contractions in Krebs solution without verapamil (Supplementary Figure 6), indicating that the Orai1-BK_Ca_ complex can negatively regulate agonist-induced small intestine contractions.

### Orai1-BK_Ca_ Interaction Alteration in Small Intestinal Smooth Muscle of Diabetic Mice

To further investigate the pathological relevance of the Orai1-BK_Ca_ interaction, we used co-immunoprecipitation assays to examine changes in the association between Orai1 and the BK_Ca_ channel. Our data indicated that compared with that in age-matched controls, the amount of BK_Ca_ protein pulled down by Orai1 was markedly decreased in the SISM of diabetic mice (Figure 5A). Western blotting analysis showed that the BK_Ca_ protein expression has not changed (Figures 5B,C). These results suggest that compared with that in control mice, the Orai1 and BK_Ca_ channel interaction in the SISM of diabetic mice is weaker.

## Discussion

Here, we examined SOCE in SISM cells cultured in HG medium or in a mouse model of type 1 diabetes. Our major findings are as follows: (1) SOCE activity and Orai1 and STIM1 expression levels in SISM cells were significantly increased in HG culture conditions or in diabetic mice compared with those in NG culture conditions or age-matched control mice, respectively; (2) gastrointestinal transit speed and SOCE-mediated contractions were markedly increased in diabetic mice; (3) siRNA knockdown of Orai1 or STIM1 rescued the augmented gastrointestinal transit speed and SOCE-mediated contractions in diabetic mice; (4) Orai1 pulled down the BK_Ca_ channel in SISM cells, and the interaction between Orai1 and the BK_Ca_ channel was decreased in diabetic mice; (5) suppressing Orai1 expression or inhibiting BK_Ca_ channel activity increased agonist-induced small intestine contractions. Thus, we demonstrated that SOCE activity and SOCE-mediated contraction were significantly increased in SISM cells cultured in HG medium or in mice with type 1 diabetes and that altered SOCE activity and Orai1-BK_Ca_ channel interactions might contribute to accelerated gastrointestinal transit speed.

Although diabetic enteropathy affects the life quality of patients with type 1 diabetes (Cheng and Yilmaz, 2015), compared with other more serious complications, patients often feel that the gastrointestinal symptoms are less likely to cause critical disabilities or death. Thus, owing to limited attention and research, the effect of type 1 diabetes on SISM is poorly understood. An imbalance in the Ca^2+^ homeostasis of muscle cells disrupts contractile function and gastrointestinal motility, and SOCE disorders may lead to many dysfunctions, ranging from immunodeficiency to myopathy and vascular diseases (Feske, 2007; Duke et al., 2010; Voelkers et al., 2010). To our knowledge, this study is the first to report the physiological mechanisms of SOCE in the SISM associated with type 1 diabetes. We found that the SOCE of MUS-M1 cells cultured in HG was increased without significantly altering Ca^2+^ store release. As expected, our western blot data showed that Orai1 and STIM1 protein expression was also significantly increased. In diabetic mice, Orai1 and STIM1 protein expression and SOCE-mediated small intestine contraction were also markedly increased, whereas high-K^+^- and CCh-induced contractions were not significantly altered. Moreover, HE staining showed that the thickness of the smooth muscle layer in the small intestine did not differ between age-matched control and diabetic mice. Suppressing Orai1 or STIM1 expression using specific siRNA did not significantly affect Ca^2+^ store release but significantly inhibited SOCE in MUS-M1 cells and SOCE-mediated small intestine contractions in diabetic and control mice without affecting high-K^+^- and CCh-induced contractions. Together these results suggest that a HG environment and diabetes enhance SOCE in SISM cells, leading to increased Ca^2+^ mobilization and small intestine contraction.

Recently, we and another group reported that Orai1 associates with the K_Ca_ channel to participate in smooth muscle contraction as well as cancer cell migration and bone metastasis (Chantome et al., 2013; Song et al., 2015; Chen et al., 2016). In the present study, we found that Orai1 also associated with the BK_Ca_ channel to regulate mouse small intestine contraction. In the flow chart of our study hypothesis, Orai1-mediated Ca^2+^ influx activates the BK_Ca_ channel, leading to membrane hyperpolarization (Supplementary Figure 7). The membrane hyperpolarization inhibits VDCCs to prevent agonist-induced small intestine contraction. Additionally, our results showed that Orai1 siRNA transfection to suppress Orai1 protein expression or IbTX administration to inhibit the BK_Ca_ channel significantly increased CCh-induced mouse small intestine contractions, but did not affect high-K^+^-induced contractions. Moreover, Orai1 protein expression and SOCE activity were increased in the SISM cells of diabetic mice. According to the working model of the Orai1-BK_Ca_ complex shown in Supplementary Figure 7, increasing Orai1 activity and SOCE should strongly activate BK_Ca_ channels, which in turn would inhibit small intestine contraction and motility largely by inhibiting VDCC opening. However, in contrast to this hypothetical model, our data indicated that small intestine transit speed was markedly increased in diabetic mice. To determine the reason for this acceleration, we investigated alterations in the Orai1-BK_Ca_ channel interaction in the SISM cells of diabetic mice. Our co-immunoprecipitation data showed that the Orai1-BK_Ca_ complex interaction was markedly weakened in the SISM cells of diabetic mice. Based on this finding, we speculate that although Orai1 protein expression and SOCE activity were increased, Orai1-mediated Ca^2+^ influx did not effectively activate BK_Ca_ channels but more likely induced muscle contraction and intestine motility. Therefore, increased Orai1 protein expression but decreased Orai1-BK_Ca_ interaction may reconcile this conflicting result. How best to alter this weakened Orai1-BK_Ca_ channel interaction in diabetic animals to achieve a therapeutic response will be examined in future studies.

Numerous previous studies have shown that the alteration of gastrointestinal tract motility in type 1 diabetes is controversial. Several studies have reported that gastrointestinal transit speed is faster in patients with type 1 diabetes than in controls (Rosa-e-Silva et al., 1996; Perano et al., 2015), but other reports indicate delayed gastrointestinal activity in these patients (Zhao et al., 2006; Faria et al., 2013). Conflicting results are also found in rodent models of type 1 diabetes (Martin et al., 2004; Izbeki et al., 2008; Umathe et al., 2009; Durmus-Altun et al., 2011; Hu and Feng, 2012). Our data indicated that gastrointestinal transit speed was markedly increased in a mouse model of type 1 diabetes. Suppressing Orai1 and STIM1 with siRNA-specific transfections reduced the gastrointestinal transit speed in diabetic mice to that of control mice. Zheng et al. (2018) reported that SOCE-mediated Ca^2+^ influx is necessary to maintain interstitial cells of Cajal (ICC) pacemaker activity. Besides, colonic pacemaker ICC-SM exhibit complex Ca^2+^-firing patterns and drive smooth muscle activity and overall colonic contractions (Baker et al., 2021). Maintenance and refilling of cellular Ca^2+^ stores by SOCE may also be important for mediation and shaping of Ca^2+^ signals in ICC-SM. Therefore, increased SOCE activity and Ca^2+^ mobilization in SISM, ICC cells and/or even nervous system may all contribute to the accelerated gastrointestinal transit speed and diabetes-associated diarrhea because in body Orai1 and STIM1 siRNAs may affect many cell types involving gastrointestinal transit speed. However, which cell is more important and why the gastrointestinal transit speed was shown to be faster in some studies but delayed in others in animal or patients with type 1 diabetes is unknown. Perhaps factors such as the stage of type 1 diabetes, meals, degree of blood glucose, etc., may differentially affect gastrointestinal transit speed. This issue should be addressed in future studies.

Store-operated Ca^2+^ entry regulates various cellular functions, including cell proliferation, contraction, and secretion (Parekh and Putney, 2005). In type 1 diabetes, morphological changes and biomechanical remodeling occur in the small intestine, manifesting as proliferation in all layers of gastrointestinal wall, increasing cross-section thickness and length and altering opening angles and residual strain (Domenech et al., 2011; Liu et al., 2012). In addition to muscle contraction, SOCE is also involved in muscle proliferation, including that of cardiomyocytes, aortic smooth muscle cells, and bronchial smooth muscle cells (Sweeney et al., 2002; Berra-Romani et al., 2008; Luo et al., 2012). To exclude this effect on the small intestine transit speed, we compared high-K^+^-induced small intestine contractions between diabetic and age-matched control mice and found no significant difference between them. Our HE staining results also showed that the SISM layer thickness was not altered in diabetic mice. Therefore, our results exclude the effect of proliferation or hypertrophy of SISM cells on enhanced small intestine transit speed.

In live cells, Ca^2+^ homeostasis regulation and Ca^2+^-mediated signal transduction are complicated. Although we demonstrated that the SOCE-mediated Ca^2+^ signal was increased in the SISM cells of mice with type 1 diabetes, another group found that Ca^2+^-activated Cl^–^ channels also participate in glucose-induced [Ca^2+^]_i_ increase in small intestine cells (Yin et al., 2014). In diabetes, the body is under a high oxidative state. Excessive reactive oxygen species can induce numerous cellular disorders, destroying Ca^2+^ homeostasis. Therefore, our finding may be only one of the pathways involved in type 1 diabetes pathology.

## Conclusion

We demonstrated that SOCE activity was significantly increased in SISM cells cultured in HG medium or in mice with type 1 diabetes. Upregulated SOCE promoted small intestinal muscle contractions and gastrointestinal transit speed, which may be among the factors inducing diabetic diarrhea. Therefore, the SOCE pathway likely represents a potential therapeutic target for diabetic diarrhea.

## Data Availability Statement

The original contributions presented in the study are included in the article/Supplementary Material, further inquiries can be directed to the corresponding authors.

## Ethics Statement

The animal study was reviewed and approved by the Animal Ethics Committee of Anhui Medical University.

## Author Contributions

FD and JG performed the experiments, analyzed the data, and co-wrote the manuscript. YW, TJ, HC, and YH conducted the experiments and analyzed the data. XX and JD designed a part of the study and helped to interpret the part of the data. BS and QZ conceived the idea, designed and supervised the study, analyzed the data, and revised the manuscript. All authors reviewed the report and approved the final version.

## Conflict of Interest

The authors declare that the research was conducted in the absence of any commercial or financial relationships that could be construed as a potential conflict of interest.

## Publisher’s Note

All claims expressed in this article are solely those of the authors and do not necessarily represent those of their affiliated organizations, or those of the publisher, the editors and the reviewers. Any product that may be evaluated in this article, or claim that may be made by its manufacturer, is not guaranteed or endorsed by the publisher.
