# Supplementary material for: Enhanced Store-Operated Ca2+ Signal of Small Intestinal Smooth Muscle Cells Accelerates Small Bowel Transit Speed in Type 1 Diabetic Mouse
Source: Front Physiol. 2021 Oct 20;12:691867. doi: 10.3389/fphys.2021.691867 (PMC8564290; doi:10.3389/fphys.2021.691867)
Supplement: Supplementary file 1 [file Data_Sheet_1.PDF]

Supplementary information for

Enhanced Store-operated Ca^{2+} Signal of Small Intestinal Smooth Muscle Cells Accelerates Small Bowel Transit Speed in Type 1 Diabetic Mouse

Fang Dai, *et al*.

Supplementary Figure 1

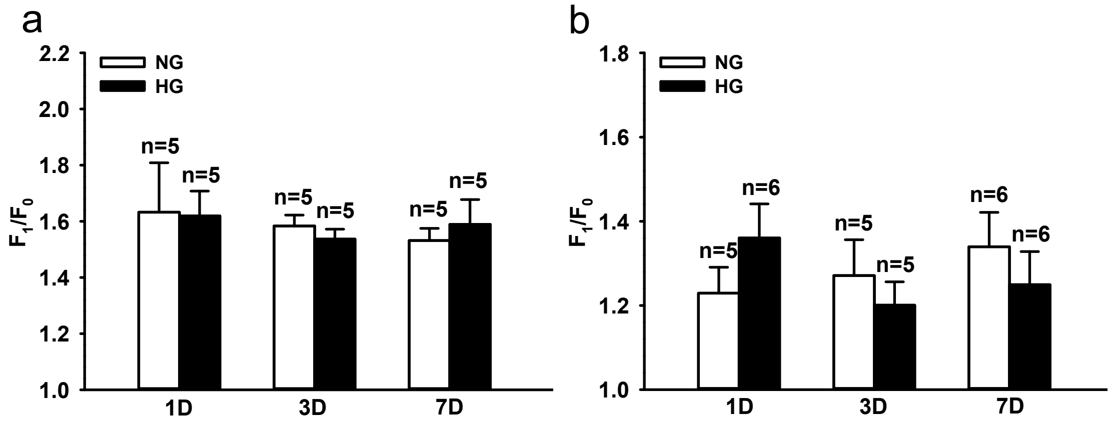

Supplementary Figure 1. Thapsigargin (TG) and carbachol (CCh)-induced intracellular Ca^{2+} ($[Ca^{2+}]_i$) rise in MUS-M1 cells. **a** and **b**, Summarized data showing TG (A)- and CCh (B)-induced [Ca2+]i rise in MUS-M1 cells cultured in normal glucose (5.6 mM D-glucose + 20 mM α-mannitol, NG) or HG (25 mM D-glucose) medium for 1, 3, and 7 days. Values are shown as the mean ± SEM (n = 5-6); $P > 0.05$, means non-significant, NG vs. HG at the same day.

Supplementary Figure 2

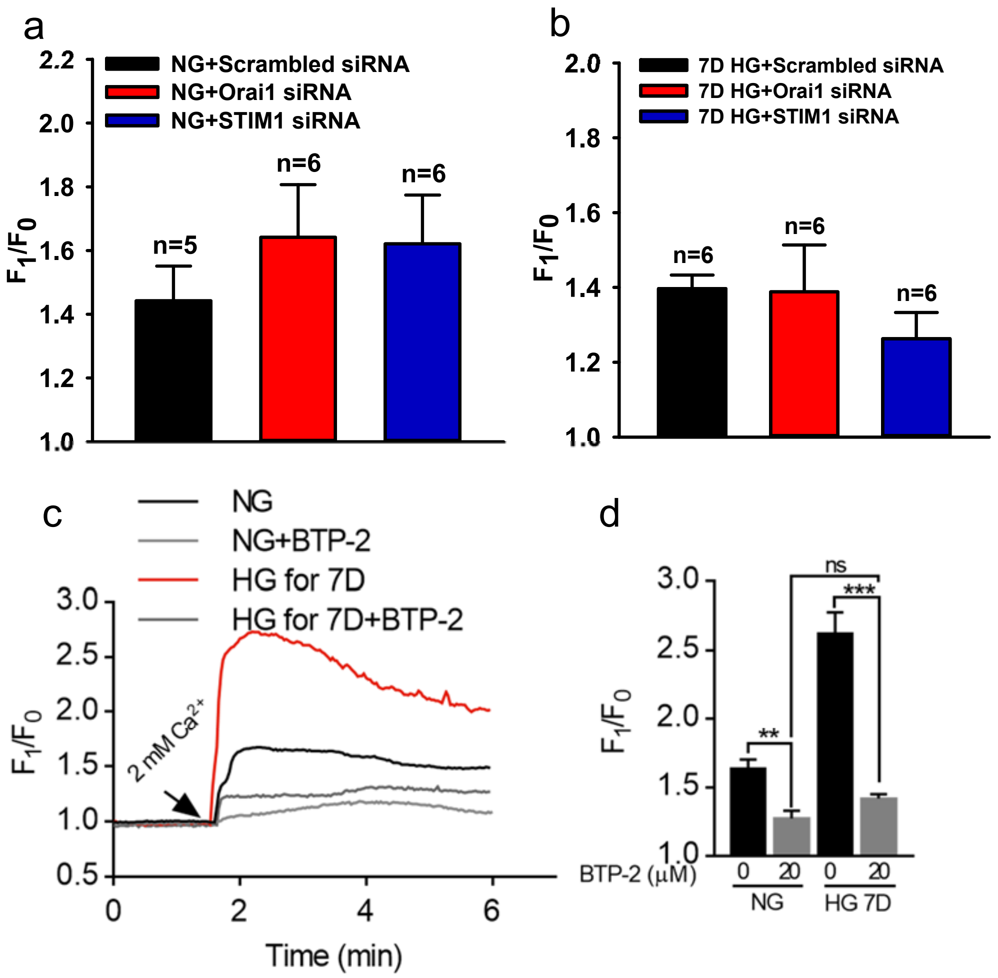

Supplementary Figure 2. Carbachol (CCh)-induced intracellular Ca^{2+} ($[Ca^{2+}]_i$) rise and the impact of BTP2 on calcium influx in MUS-M1 cells. **a**, Summarized data showing CCh-induced $[Ca^{2+}]_i$ rise in Ca^{2+}-free solution in MUS-M1 cells transfected with Orai1, STIM1 or scrambled siRNA. **b**, Summarized data showing CCh-induced $[Ca^{2+}]_i$ rise in Ca^{2+}-free solution in MUS-M1 cells cultured in high glucose (HG, 25 mM D-glucose) medium for 7 days and transfected with Orai1, STIM1 or scrambled siRNA. Values are shown as the mean ± SEM (n = 5-6); $P > 0.05$, means non-significant, scrambled siRNA vs. Orai1 or STIM1 siRNA transfection. **c, d**, Representative traces (**c**) and summarized data (**d**) showing the alterations in $[Ca^{2+}]_i$ (SOCE) evoked by extracellular application of 2 mM Ca^{2+} after the treatment of 100 μM carbachol for 10 min in Ca^{2+}-free solution to deplete internal Ca^{2+} stores in MUS-M1 cells cultured in normal and high glucose medium with or without BTP-2 pre-incubation. Values are shown as the mean ± SEM; **, and *** denote $P < 0.01$, and $P < 0.001$, respectively. NS means non-significant.

Supplementary Figure 3

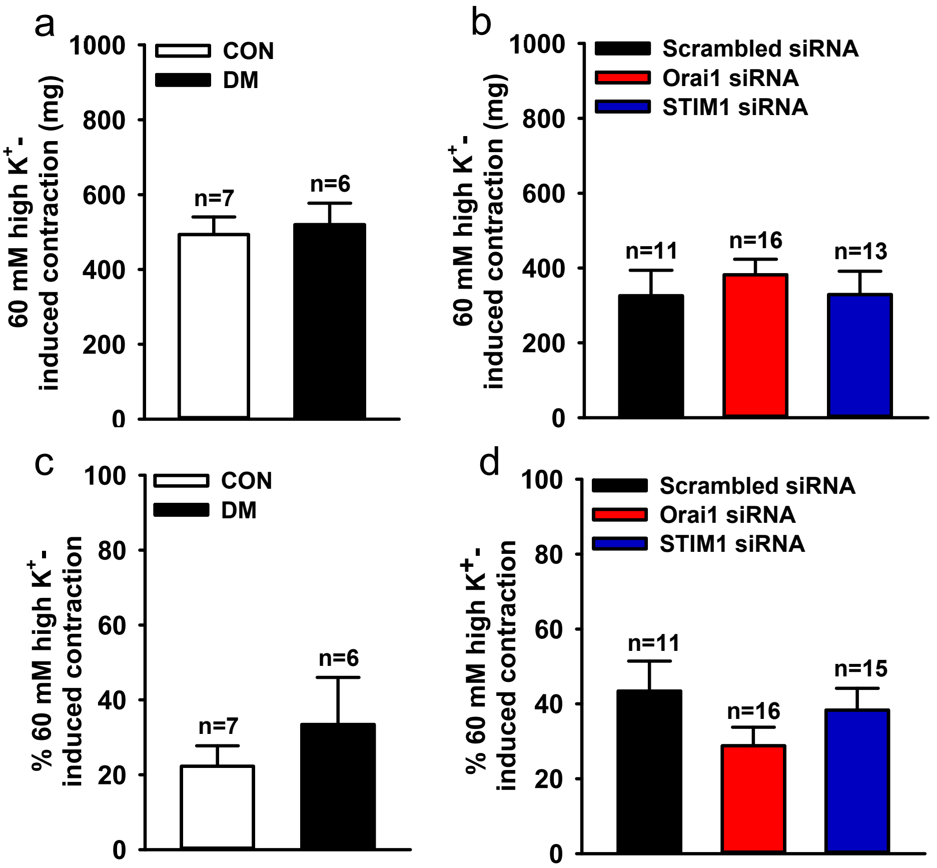

Supplementary Figure 3. 60 mM high K^+ solution- and carbachol (CCh)-induced mice small intestinal longitudinal contraction and effect of Orai1 and STIM1 siRNA transfection. **a** and **b**, Summarized data showing 60 mM high K^+ solution-induced mice small intestinal longitudinal contraction in age control (CON) and diabetic mice (DM) (**a**), or diabetic mice transfected with Orai1, STIM1 and scrambled siRNAs (**b**) in Krebs solution, in which 60 mM (high) K^+-induced contraction is caused by voltage-dependent Ca^{2+} channel-mediated Ca^{2+} influx. **c** and **d**, Summarized data showing 100 μM CCh-induced mice small intestinal longitudinal contraction in age control (CON) and diabetic mice (DM) (**c**), or diabetic mice transfected with scrambled, Orai1 and STIM1 siRNA (**d**) in Ca^{2+}-free solution, in which CCh-induced contraction is induced by Ca^{2+} release from Ca^{2+} store. Values are shown as the mean ± SEM (n = 6-16); $P >$ 0.05, means non-significant, age control (CON) vs. diabetes (DM), scrambled siRNA vs. Orai1 or STIM1 siRNA transfection.

Supplementary Figure 4

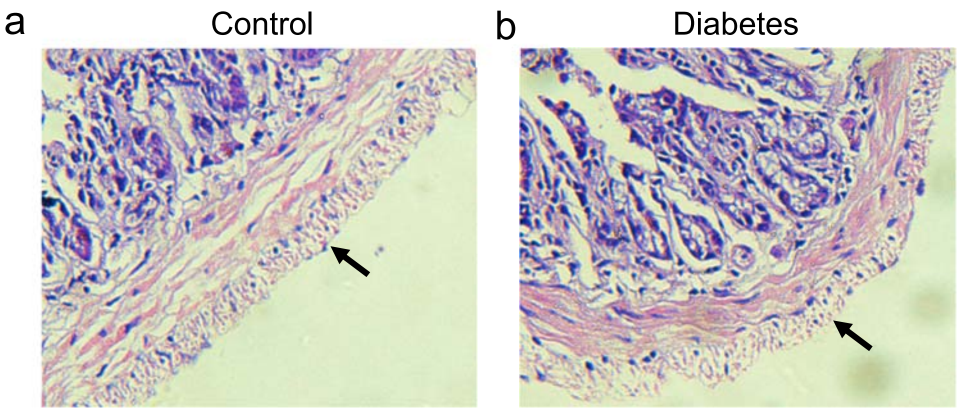

Supplementary Figure 4. Hematoxylin and eosin (HE) staining of mice small intestinal section. Representative images showing the thickness of longitudinal smooth muscle cell layer of age control (**a**) and diabetic mice (**b**). Arrows indicate longitudinal smooth muscle cell layer.

Supplementary Figure 5

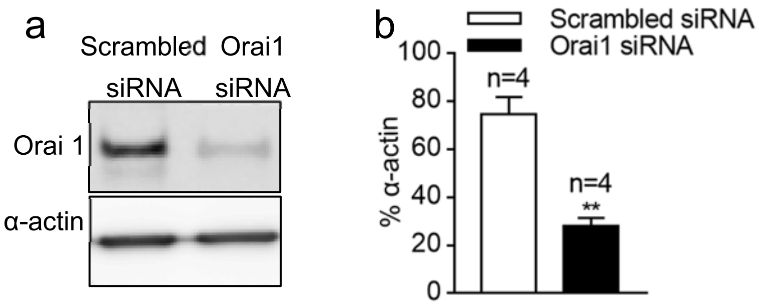

Supplementary Figure 5. Effect of Orai1 siRNAs on Orai1 expression in normal mice small intestinal smooth muscle cells. Representative immunoblotting images and summarized data showing Orai1 expression levels in normal mice small intestinal smooth muscle cells transfected with Orai1 siRNA or scrambled control siRNA (**a, b**). Alpha-actin was used as a loading control. Values are shown as the mean ± SEM (n = 4); $^{**}P < 0.05$, scrambled siRNA vs. Orai1 siRNA transfection.

Supplementary Figure 6

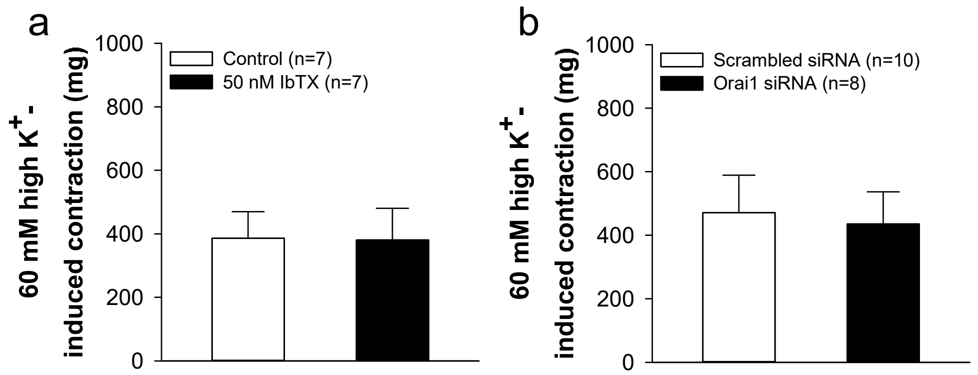

Supplementary Figure 6. 60 mM high K^+ solution-induced small intestinal segment longitudinal contraction in normal mice. The mice small intestinal segment was pretreated by 50 nM iberiotoxin (IbTX, **a**) for 20 min or transfected with Orai1 siRNA (**b**). Summarized data showing 60 mM high K^+ solution-induced small intestinal segment longitudinal contraction in Krebs solution without verapamil. Values are shown as the mean ± SEM (n = 7-10); $P > 0.05$, means non-significant, control vs. IbTX treatment.

Supplementary Figure 7

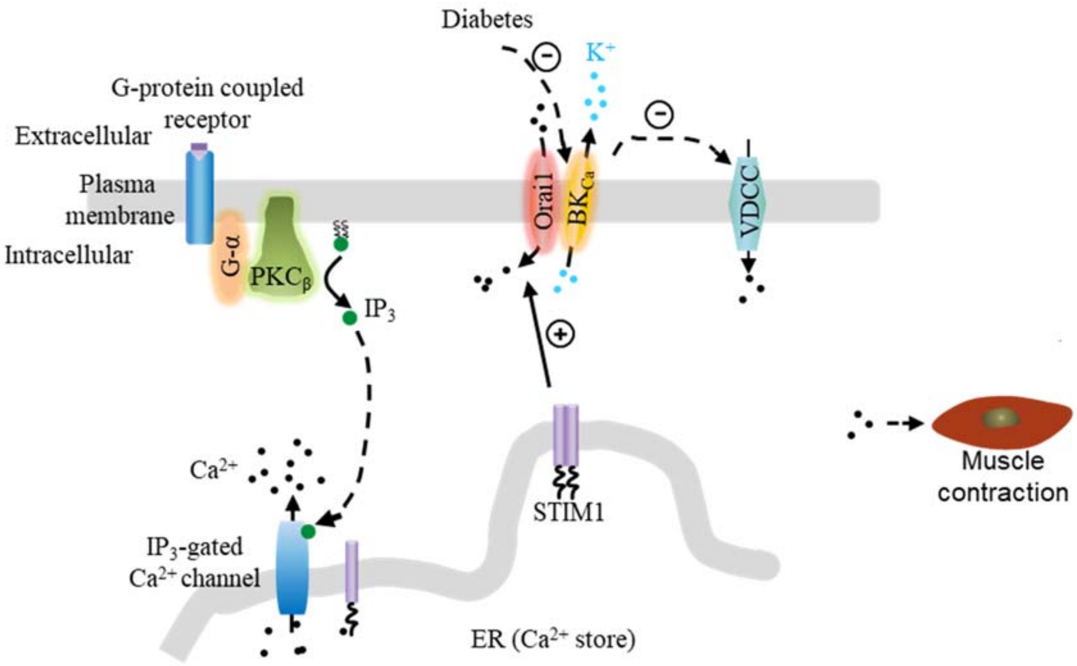

Supplementary Figure 7. Scheme of signal pathway in our study. The scheme showing the working mechanisms of store-operated Ca^{2+} entry and Orai1-BK_{Ca} complex. The activation of G protein-coupled receptors (GPCRs) causes phospholipase C_β to convert phosphatidylinositol 4,5-biphosphate into IP3 and diacylglycerol. The IP3 activates IP3R induce the Ca^{2+} release. Depletion of ER Ca^{2+} causes STIM1 to polymerize oligomerize to form puncta. The polymerized C-terminus of STIM1 in turn activates Orai1 to mediate SOCE. Orai1-mediated Ca^{2+} influx activates the BK_{Ca} channel, leading to membrane hyperpolarization, the membrane hyperpolarization inhibits VDCCs to prevent agonist-induced small intestine contraction. Orai1 and BK_{Ca} channel interaction in the SISM of diabetic mice is weaker. G-α, guanine nucleotide binding protein α-subunit; phospholipase C beta, PLCβ; IP_3, inositol triphosphate; IP_3R, inositol triphosphate receptor/IP_3-gated Ca^{2+} channel; ER, endoplasmic reticulum; STIM1, stromal interaction molecule 1; BK_{Ca}, larger conductance Ca^{2+}-activated K^+ channels; VDCC, voltage-dependent Ca^{2+} channels.
